# Envelhecer nas prisões

**DOI:** 10.1590/0102-311XPT255025

**Published:** 2026-05-29

**Authors:** Natália Vargas Fialho, Martinho Braga Batista e Silva

**Affiliations:** 1 Universidade do Estado do Rio de Janeiro, Rio de Janeiro, Brasil.

Em dez capítulos, contendo dados quantitativos e qualitativos recolhidos entre os anos de 2019 e 2020, reunindo desde notas de um trabalho de campo até os resultados da aplicação de questionários em 682 das 763 pessoas enclausuradas em 38 estabelecimentos penais no Estado do Rio de Janeiro, Brasil, Maria Cecília de Souza Minayo & Patrícia Constantino ^1^ iluminam uma temática sensível, a respeito de um segmento populacional que reúne múltiplas vulnerabilidades: pessoas que atravessam o processo de envelhecer, com seus problemas odontológicos, oftalmológicos, ortopédicos e neurológicos, e se encontram encarcerados em espaços insalubres, superlotados e com precária alimentação.

Foi feito um trabalho valioso de reunir uma série de políticas de proteção, abarcando as categorias “idoso” e “privado de liberdade”, como a Política Nacional de Atenção Integral à Saúde das Pessoas Privadas de Liberdade no Sistema Prisional (PNAISP), a Lei de Execução Penal e o Estatuto do Idoso, além de apresentar comparativos por gênero, classe social, nível de escolaridade e políticas de outros países. Entretanto, o estudo não chega a mencionar a Política Nacional de Saúde Ocular, vigente no Brasil desde 2008, embora grande parte dos participantes da pesquisa apresente importantes problemas oftalmológicos: 73,85% possuem “defeito da visão” e 13,94% apresentam cegueira em um ou ambos os olhos (em comparação a 3% na população brasileira geral) ^1^. Estes dados convocam uma nova categoria: a deficiência.

A cartilha *Procedimentos Direcionados às Pessoas com Deficiência no Sistema Prisional* ^2^ evoca diretrizes nacionais e internacionais, como o Estatuto da Pessoa com Deficiência, e indica que as pessoas com deficiência não devem ser submetidas a tortura, a tratamentos ou penas cruéis, desumanos ou degradantes e, quando privadas de liberdade, a prisão deve estar em conformidade com a lei. Além disso, os espaços devem ser acessíveis, ajustados para garantir a plena participação nas atividades, o que não é encontrado na pesquisa. O documento indica ainda a existência de uma Comissão Técnica de Classificação responsável por investigar possíveis especificidades quanto às deficiências e alocar a pessoa presa com deficiência em espaço específico, respeitando a acessibilidade ou adaptação razoável, o que não é visto na pesquisa das autoras.

O livro apresenta os caminhos de pensamento da pesquisa, que inclui um inquérito sobre saúde e qualidade de vida dos idosos presos e custodiados; entrevistas com idosos presos e custodiados, com gestores de unidades prisionais e com profissionais que trabalham diretamente com esse público; observação sistemática da realidade da prisão registrada em diário de campo; e, por fim, um rastreio cognitivo por meio do MEEM (*Mini-exame do Estado Mental*), também conhecido como Minimental.

Sobressai aos olhos o tratamento dado ao objeto da pesquisa, que não se restringe àqueles privados de liberdade, como se fossem alheios ao mundo e vice-versa, mas dialoga com trabalhadores, gestores e inclui o olhar extramuro dos pesquisadores sobre o ambiente prisional, relatando, com riqueza, as chocantes impressões daqueles que não habitam tal local em seus cotidianos.

Aspectos como o perfil sociodemográfico e criminal dos participantes também foram apontados, destacando que a maior parte das pessoas idosas privadas de liberdade são homens (94,5%), quase metade é casada (46,8%), a maioria tem, em média, aproximadamente quatro filhos (92,7%), se declara parda (43,9%) e relata praticar alguma religião às vezes ou frequentemente (75%). Destaca-se ainda o grau de escolaridade, onde 10,5% completaram o ensino médio e 6,9% possuem ensino superior completo (geralmente políticos, agentes de segurança e servidores presos), sendo que esta última taxa para a população prisional não idosa é de 1,5% ^3^.

Finalmente, chama atenção o fato de a maior parte dos homens idosos terem cometido crimes sexuais, enquanto a maior parte dos homens encarcerados lá, estão por crimes contra o patrimônio ou a vida. Inclusive, durante a pandemia de COVID-19, período posterior à pesquisa, as recomendações de desencarceramento desse segmento populacional por parte do Conselho Nacional de Justiça encontraram limitações por conta dessa tipificação penal hegemônica: “*A questão dos idosos, por exemplo: teve uma época que ficou ‘vão sair’, ‘não vão sair’... falando bem honestamente, o problema da população idosa dentro do sistema penitenciário é que muitos deles são condenados por crimes sexuais. Essa é a grande questão, sempre se esbarra nisso*” ^4^ (p. 66).

Podemos questionar: por que fazer uma pesquisa que se concentre nessa faixa etária, uma vez que diversas pesquisas já demonstraram o efeito danoso do ambiente prisional em todas as faixas etárias? Para quem caminha pelas páginas deste livro, a resposta é facilmente colocada. O ambiente carcerário não é adequado às necessidades dos presos idosos, não há acompanhamento médico, odontológico e farmacêutico como prevê a Lei de Execução Penal, sendo que a maior parte dos idosos apresenta, principalmente, problemas dentários, com desdobramentos significativos na dificuldade de alimentação, bem como demandas de medicamentos invariavelmente indisponíveis no sistema prisional.

Atividades básicas para o público em questão, como alimentação (necessita de alimentos nutritivos e dentição apropriada), deslocamento até o banheiro (fundamental para sujeitos com perda urinária ou fecal), leitura (única forma de lazer geralmente disponível, incluindo atividades religiosas), dentre outras atividades, são praticamente inviáveis nas prisões fluminenses. Um dos presos relata: “*Só estou emagrecendo. Não tenho dentes e a comida é crua e dura*” ^1^ (p. 144), enquanto outro comenta: “*...O banheiro é uma privada* [!] *para duzentas pessoas, não tem um lugar para se apoiar*” ^1^ (p. 145). São inúmeros relatos das múltiplas punições que se unem à punição da privação de liberdade, como de um entrevistado de 81 anos que diz “*Escreve aí que estou morrendo*” ^1^ (p. 213).

A solidão, sentimento amplamente mencionado quando o assunto é o envelhecimento, é acentuada no cárcere, uma vez que muitos idosos carregam o estigma da acusação por um crime, não recebem visitas e encontram-se desamparados, dispersos no conjunto total que une todas as idades, sem possibilidade de amparo familiar, social ou do Estado, sem assistência, medicação ou alimentação digna. As autoras destacam esse quadro em mulheres presas, reservando um capítulo inteiro sobre o assunto. Surgem questões importantes, como a correlação entre os sintomas apresentados pelas idosas e a presença ou ausência de visitação, com altos índices de angústia/ansiedade (76,9% das que recebem visitas e 93,3% das que não recebem).

Ao realizarem uma etnografia nas filas de espera de duas penitenciárias femininas, Lermen & Silva ^5^ corroboram o apontado, afirmando que a quantidade de visitantes é significativamente menor que nas prisões masculinas e, ainda assim, a maioria das visitas a mulheres é realizada por outras mulheres, destacando como o cuidado ainda é generificado.

Na população de presas e presos idosos, cabe ressaltar que as autoras não apontam somente as particularidades da permanência na prisão, mas também as especificidades requeridas no processo de ressocialização, paralelamente insuficiente. Os desafios encontrados no cuidado com idosos em liberdade se alargam e cronificam para o público em questão: abandono ou solidão, difícil acesso a direitos como moradia e renda mínima, indisponibilidade de instituições públicas de cuidado, como instituições de longa permanência para idosos (ILPI) e de atividades acessíveis de lazer, socialização e trabalho, quando aplicável. A ressocialização que não ocorre com jovens, ocorre menos ainda com os maiores de 60 anos.

A obra reúne dados impressionantes junto à participação e descrição dos afetos despertados nos pesquisadores diante de um cenário pouco visibilizado e de extrema fragilidade. Diferente de muitas pesquisas, esta torna os dados em produto: a busca ativa pelos direitos dessa população, através de documentos, solicitações formais e convocação da sociedade para enfrentar esse grave problema. Essa é uma oportunidade ímpar para conhecer os frágeis e invisíveis.
